# Microstructure and Thermal Property of Designed Alginate-Based Polymeric Composite Foam Materials Containing Biomimetic Decellularized Elastic Cartilage Microscaffolds

**DOI:** 10.3390/ma15010258

**Published:** 2021-12-30

**Authors:** Ching-Cheng Huang

**Affiliations:** 1 Department of Biomedical Engineering, Ming-Chuan University, Guishan District, Taoyuan 320-33, Taiwan; junas.tw@yahoo.com.tw; 2 PARSD Biomedical Material Research Center, Xitun District, Taichung 407-49, Taiwan

**Keywords:** supercritical fluid, decellularized extracellular matrix, elastic cartilage, alginate, foam

## Abstract

This study presents a designed alginate-based polymeric composite foam material containing decellularized elastic cartilage microscaffolds from porcine elastic cartilage by using supercritical fluid and papain treatment for medical scaffold biomaterials. The microstructure and thermal property of the designed alginate-based polymeric composite foam materials with various controlled ratios of alginate molecules and decellularized elastic cartilage microscaffolds were studied and characterized by Fourier transform infrared spectroscopy (FTIR), scanning electron microscopy (SEM), and differential thermal gravimetric analysis (TGA/DTG). The microstructure and thermal property of the composite foam materials were affected by the introduction of decellularized elastic cartilage microscaffolds. The designed alginate-based polymeric composite foam materials containing decellularized elastic cartilage microscaffolds were ionically cross-linked with calcium ions by soaking the polymeric composite foam materials in a solution of calcium chloride. Additional calcium ions further improved the microstructure and thermal stability of the resulting ionic cross-linked alginate-based polymeric composite foam materials. Furthermore, the effect of crosslinking functionality on microstructures and thermal properties of the resulting polymeric composite foam materials were studied to build up useful information for 3D substrates for cultivating and growing cartilage cells and/or cartilage tissue engineering.

## 1. Introduction

Osteoarthritis (OA) is a common disease of the elderly worldwide, which is characterized by articular cartilage destruction and local inflammation, resulting in pain, disability and a significantly reduced quality of life for the affected individuals. Numerous polymeric materials such as synthetic materials and natural materials, including sodium alginate, fibrin, collagen, nanocellulose, chitosan, starch, poly(lactic acid), and polycaprolactone had been proposed, modified, and used for 3D bioinks or medical applications such as those for osteoarthritis [1,2,3,4,5,6,7,8,9,10,11,12,13,14,15,16,17]. Some biomaterials may not be suitable for use with cells due to their physical and structural configuration. Alginate is a natural polysaccharide exhibiting excellent biocompatibility, biodegradability, and chelating ability, having many different applications in the biomedical fields. Sodium alginate is a naturally occurring biopolymer extracted from different species of marine brown algae [13,15,16,17]. Alginate is readily processable for applicable three-dimensional scaffolding materials such as hydrogels, microspheres, microcapsules, sponges, foams, and fibers. Alginate-based biomaterials can be utilized as drug delivery systems, for cell immobilization, and as cell carriers for tissue engineering. Alginate can be easily modified via chemical and physical reactions to obtain derivatives having various structures, properties, functions, and applications [13,15,16,17]. Liu et al. reported that calcium alginate membrane with 5 wt% CaCl_2_ for matrix of protein molecular-imprinted membrane promoted cell adhesion and proliferation [17]. In addition to bioinks, acellular materials were used in 3D bioprinted structures. Acellular materials typically provide structural support for tissue constructs and, when utilized with bioinks, can generate functional bioprinted tissues. Acellular materials can provide porous microstructures that recapitulate both mechanical and biochemical properties of the native extracellular matrix. Porosity enables cell migration, tissue growth, vascular formation, and cell viability within the microstructural constructs. In addition, acellular materials must also have the necessary surface chemistry for cell attachment, cell proliferation, and cell differentiation. Additionally, alginate-based biomaterials can be used as a 3D culture matrix because the biomaterials can provide support for the integration of cells and can act as a platform for cellular growth. Alginate-based biomaterials have the ability to form scaffolds or hydrogels in the presence of divalent ions (such as calcium ions) because of the carboxyl groups in the polymer chains. Decellularized extracellular matrix scaffolds have a lot of collagens, which constitute the main structural element of the decellularized extracellular matrix, provide tensile strength, regulate cell adhesion, support migration, and direct tissue development [18,19,20,21]. The objectives of the present manuscript were to provide a design of composite foam materials containing alginate and decellularized extracellular matrix with high stability and their expected effects on the biological properties of the scaffolds [17,18,19,20]. Lee et al. reported on the design and preparation of new bone-derived decellularized extracellular matrix/alginate bioink with a specific decellularized extracellular matrix/AG ratio less than 50% that was employed to build up a 3D cell-laden mesh structure for bone tissue engineering [22]. In previous work, a series of amphiphilic polymers and polybetaines were designed and prepared for biomedical potential applications [1,7,8,9,10,11,12]. Recently, natural materials such as carbodiimide crosslinked biodegradation-controllable small intestinal submucosa (SIS) sheets were prepared, which exhibited a good regenerative potential for soft tissue repair [18]. Moreover, new decellularized fibrous microscaffold and corresponding alginate-based composite scaffold membranes were prepared from porcine skin by using a designed decellularization procedure such as plant enzymes/aqueous two-phase method and supercritical fluid [2,23]. The low hemolysis percentage of the decellularized scaffolds derived from porcine tissue was observed and demonstrated good antihemolytic characteristics [24].

In this study, new decellularized elastic cartilage microscaffolds (dECmS) were prepared by using a designed decellularization procedure that combined supercritical carbon dioxide with specific enzymes with the aim of their use in osteoarthritis treatments and cartilage regeneration. A series of new composite foam materials containing dECmS and alginate (AG) were prepared. For clinic applications, the microstructures and thermal properties of the biomaterials are important. The effect of crosslinking functionality on microstructures and thermal properties of the resulting polymeric composite foam materials (CFM) containing dECmS with various dECmS/AG ratios less than 50%, such as 5/95, 15/85, and 20/80, was studied to build up useful information for 3D bioprinting as 3D substrates for cultivating and growing cartilage cells and/or medical application such as osteoarthritis and cartilage regeneration for cartilage tissue engineering.

## 2. Materials and Methods

### 2.1. Materials

**Scheme 1:**
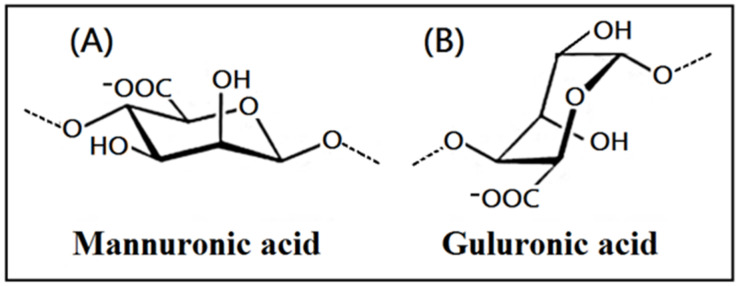
The chemical structure of alginate containing mannuronic acid (**A**) and guluronic acid (**B**).

The chemicals used in the study, such as sodium alginate, TritonX-100, NH_4_OH, NaOH, acetic acid, alcohol, disodium hydrogen phosphate, and calcium chloride (Scheme 1) were purchased from Sigma-Aldrich Company (Saint Louis, MO, USA).

### 2.2. Treatments with Supercritical Carbon Dioxide (ScCO_2_)

Considering medical application and gaining ISO13485 quality certification, a steady thickness of about 0.1 mm of porcine elastic cartilage sheet was obtained by using a designed tissue-cutting machine (Taiwan PARSD Pharm. Tech. Consulting Ltd. Co., Taichung, Taiwan). Supercritical carbon dioxide (ScCO_2_) was used for preparation of designed microscaffolds in this study. The ScCO_2_ was employed before decellularization treatments for removing most fatty acids and tissues. The resulting porcine elastic cartilage sheet was placed in a tissue holder, which was then placed into a ScCO_2_ vessel system. The ScCO_2_ system was then operated at 350 bar and 35 °C for 2 h [23]. The ScCO_2_ fluids-pretreated porcine elastic cartilage sheet was obtained.

### 2.3. Preparation of Designed Elastic Cartilage Microscaffolds

After treating the samples of porcine elastic cartilage sheet with ScCO_2_, the samples were soaked in 25 wt% NH_4_OH_(aq)_ for 2 h with a magnet mixer and treated with an aqueous solution of 0.05 U/mL papain at 25 °C for 2 h. The resulting samples were washed with 25% ethanol under ultrasonic wave for 1 h to remove residual fat and organic matter, frozen for 6 h, and lyophilized (EYELA, FD-5N) overnight at 0.1–0.2 torr at a freeze-drying temperature of −45 °C. A ScCO_2_ fluids-decellularized elastic cartilage microscaffold (dECmS) sheet was obtained. After micronization, micronized powders of the resulting designed elastic cartilage microscaffolds were obtained.

### 2.4. Preparation of Alginate-Based Composite Foam Materials Containing dECmS

A series of alginate-based composite foam materials with dECmS were prepared based on various ratios of dECmS and alginate (AG). Briefly, the desired amount of dECmS powder was first dispersed completely in 40 mL of double distilled water with the homogenizer at 26,000 rpm for 3 min. Then, aqueous AG solution was homogenized thoroughly with the dispersed dECmS solution at 26,000 rpm for 3 min. The aqueous dECmS/AG solutions with a fixed solid/solution ratio (*w/v*) of 5/50 were then molded and frozen for 6 h and then lyophilized (EYELA, FD-5N) overnight with the use of a freeze dryer at 0.1–0.2 torr at a freeze-drying temperature of −45 °C. A series of AG-based polymeric composite foam materials with dECmS was obtained, such as PCFM1N, PCFM2N, and PCFM3N (Table 1).

### 2.5. Preparation of Cross-Linked AG-Based Composite Foam Materials Containing dECmS

The 2 g of AG-based polymeric composite foam materials with dECmS was further soaked in 20 mL of 5% aqueous calcium chloride (CaCl_2_) solution for crosslinking reaction with magnet mixer. The cross-linked AG-based polymeric composite foam materials with dECmS was then frozen and dried by the same procedure described above. A series of designed cross-linked AG-based polymeric composite foam materials with decellularized elastic cartilage microscaffolds such as PCFM1H, PCFM2H, and PCFM3H was obtained (Table 1).

### 2.6. Measurements

Fourier transform infrared (FTIR) spectra were recorded with a spectrometer (Nicolet IS10, Thermo Fisher, Madison, WI, USA) using KBr discs and collecting data from 400 to 4000 cm^−1^. Thermal analysis was performed by thermogravimetry analysis (TGA) using a thermoanalyzer (7300TG/DTA, Seiko, Japan). All measurements employed a linear heating rate of 10 °C min^−1^, nitrogen as carrier gas, and a platinum empty pan as reference material and collected data from 50 to 550 °C. Microscaffolds were studied by scanning electron microscopy (SEM) (S3400N, Hitachi, Japan).

## 3. Results and Discussion

### 3.1. Characterization of New Elastic Cartilage Microscaffolds

**Figure 1:**
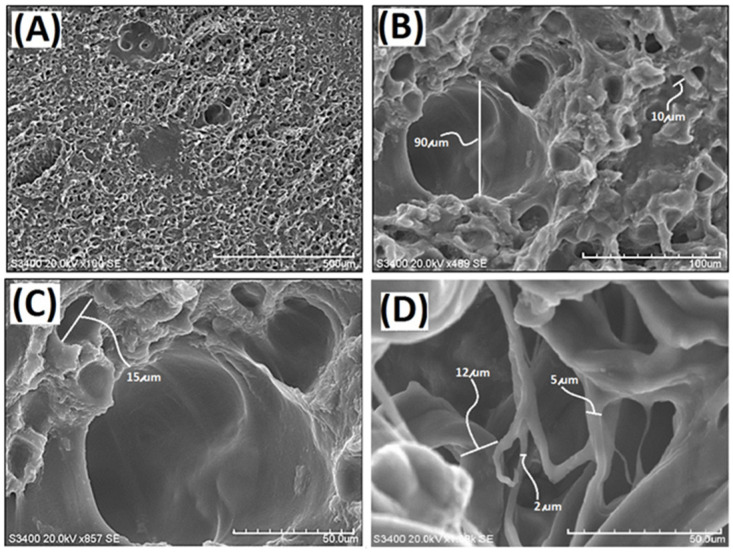
Scanning electron micrographs of the decellularized elastic cartilage microscaffold, PCFM0N: (**A**) PCFM0N membrane (scale bar 500 μm), (**B**) PCFM0N membrane (scale bar 100 μm), (**C**) PCFM0N membrane (scale bar 50 μm), and (**D**) PCFM0N powder (scale bar 50 μm).

In this study, a decellularized elastic cartilage microscaffold, dECmS, was prepared by using ScCO_2_ technology. The microstructure of resulting decellularized elastic cartilage microscaffold, dECmS, was characterized by scanning electron microscope (SEM). Scanning electron micrographs of dECmS sample are shown in Figure 1.

The hole microstructure was observed in the PCFM0N derived from porcine elastic cartilage, which was quite different from the fibrous microstructures of microscaffolds from porcine skin pretreated by supercritical carbon dioxide [23]. The averaged hole-size was found in a range of 10–20 µm (Figure 1). After micronization, PCFM0N micronized powder with a fibrous microstructure was obtained, as shown in Figure 1D. The diameter of the fibrous microstructure was observed in a range of 2–12 µm. A new microscaffold material was obtained successfully.

**Figure 2:**
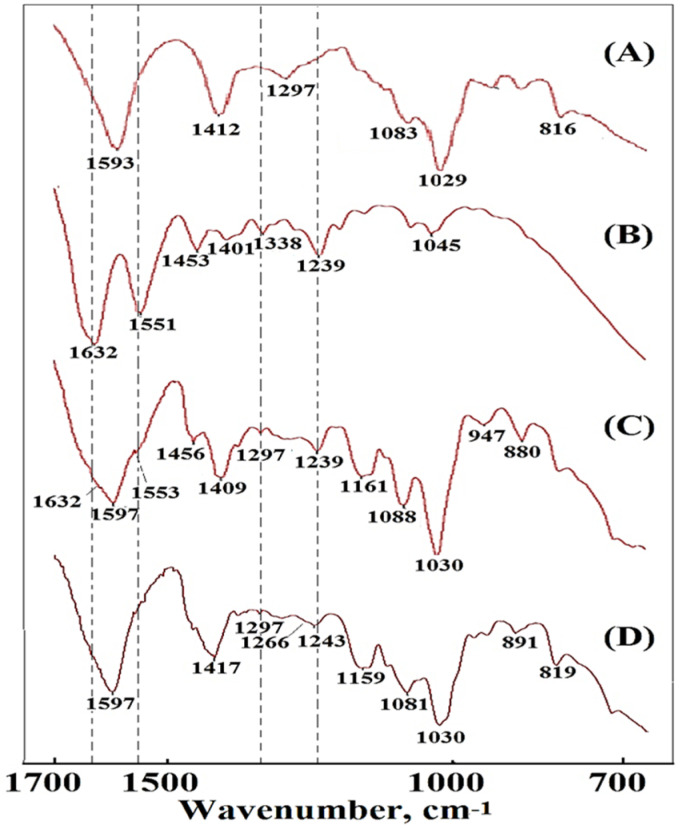
FTIR spectra of the samples in the 700–1700 cm^−1^ region: (**A**) AG, (**B**) PCFM0N, (**C**) a composite foam material PCFM3N without ionic crosslinking reaction of CaCl_2(aq)_, and (**D**) a cross-linked composite material PCFM3H with ionic crosslinking reaction of CaCl_2(aq)_.

Elastic cartilage contains elastic fibers as well as collagen and proteoglycan (primarily aggrecan). In the spectrum of dECmS (Figure 2B), the amide A and amide B bands were centered at 3290 cm^−1^ and 3090 cm^−1^, respectively; these bands are primarily associated with the stretching vibrations of N-H groups. In addition, the collagen amide I (C=O) and amide II bands of dECmS were observed at 1632 cm^−1^ and 1551 cm^−1^, respectively. The absorption band at 1240 cm^−1^ was attributed to the amide III absorptions of collagens in the dECmS. The bands at 1338 cm^−1^ are related to the CH_2_ side-chain vibrations and the triple helical structure of collagen type II [3]. The bands at 1030–1161 cm^−1^ are related to the -C-O-C- stretching vibrations. The absorption bands at 1140–1180 cm^−1^ and 984–1140 cm^−1^ were attributed to proteoglycan and aggrecan, respectively [4].

Furthermore, hemocompatibility of decellularized scaffold was assessed by hemolysis. The hemolysis percentage denotes the degree of red blood cells broken by the test sample in contact with blood. In previous work, the low hemolysis percentage of the decellularized scaffolds derived from porcine tissue was observed, and the scaffolds demonstrated good antihemolysis characteristics [25]. Similarly, Choi et al. reported that biochemical and mechanical features of a decellularized extracellular matrix supported the adhesion and growth of human cells in vitro, and the decellularized extracellular matrix exhibited biocompatibility, long-term stability, and bioinductivity in vivo [26]. The introduction of decellularized extracellular matrix derived from porcine tissue into the materials could be useful as an alternative biomaterial for xenograft tissue engineering [26].

### 3.2. Fourier Transform Infrared Spectroscopy Analysis of Newly Designed Composite Foam Materials with Microscaffolds

In this study, sodium alginate showed a broad peak at 3437 cm^−1^ for its hydrogen-bonded OH group. Asymmetric and symmetric stretching of the C=O group of alginate was observed at 1593 and 1412 cm^−1^, respectively. In addition, sodium alginate showed a characteristic peak at 1096 and 820 cm^−1^, corresponding to C-O stretching vibration and Na-O bond vibration, which indicates that sodium alginate has a large number of random coil structures (Figure 2A).

**Figure 3:**
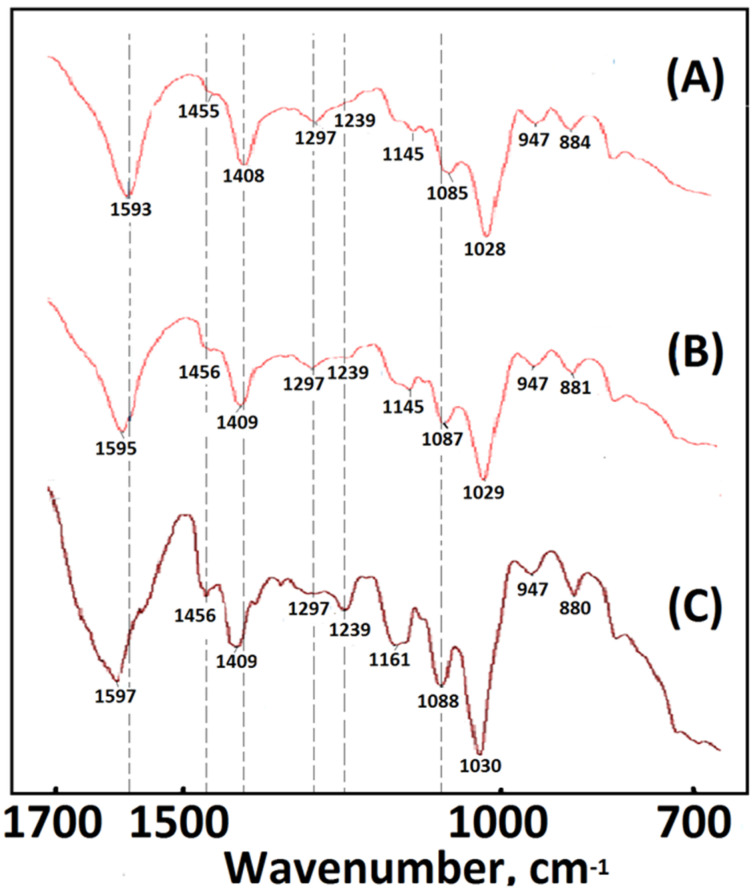
FTIR spectra of the samples: (**A**) PCFM1N, (**B**) PCFM2N, and (**C**) PCFM3N.

Furthermore, a series of alginate-based composite foam materials was prepared with various introduction ratios of AG and dECmS by using lyophilized technology. The FTIR spectroscopy profiles of the composite foam materials of AG and dECmS are shown in Figure 3. The spectra of composite foam materials of AG and dECmS with different AG and dECmS ratios had slight significant differences, as shown in Figure 3. These composite foam materials showed a wide band of -OH stretching on 3275–3280 cm^−1^: two characteristic peaks of symmetric and asymmetric stretching of -COO at 1593–1597 cm^−1^ and 1408–1409 cm^−1^, respectively. The peak of 1028–1030 cm^−1^ corresponds to the ether (Figure 3).

In the FTIR spectrum of an alginate-based polymeric composite foam material with a dECmS/AG ratio of 20/80, in addition to retaining the above-mentioned bands of alginate, there was COO symmetric stretching at 1597 cm^−1^, COO asymmetric stretching at 1417 cm^−1^, C-O stretching at 1297 cm^−1^, and C-O-C stretching at 1081 cm^−1^ and 1030 cm^−1^. In the spectrum of sodium alginate, the C-O vibration (1266 cm^−1^) of -COO- was weak, and a slight absorption peak appeared (Figure 2C). Additionally, FTIR analysis was carried out to confirm the incorporation of dECmS in the alginate-based polymeric composite foam materials (Figure 2C). The FTIR analysis of composite material containing dECmS with a dECmS/AG ratio of 20/80 showed a large overlapping stretching vibration absorption at -NH_2_ and -OH in the range of 3600–3000 cm^−1^ and -CH vibration bands in the range of 2930~2845 cm^−1^. The overlapping absorption peak of the collagen amide I (C=O) and amide II bands of dECmS and COO symmetric stretching of alginate was observed at 1597 cm^−1^. Two shoulders contributed to by collagen amide I (C=O) and amide II of the dECmS molecule were observed at 1632 cm^−1^ and 1553 cm^−1^, respectively. Furthermore, the absorption band at 1239 cm^−1^ was attributed to the amide III absorptions of collagens in the dECmS (Figure 2C). Observed bands at 1597 cm^−1^ and 1409 cm^−1^ were attributed to asymmetric and symmetric stretching vibrations of the COO- groups, respectively, and were specific to the ionic binding to form three-dimensional crosslinked networks (Figure 2C).

In this study, the resulting alginate-based polymeric composite foam materials containing dECmS were further crosslinked with CaCl_2(aq)_ to prepare the corresponding crosslinked composite foam materials, as shown in Table 1. The crosslinking functionality affected the microstructures and thermal stability of crosslinked alginate-based polymeric composite foam materials containing dECmS when the added Ca^2+^ ion penetrated into the microstructure of the composite foam materials. The cross-linking procedure with Ca^2+^ gave rise to an apparent shift of -COO symmetric stretching vibration from 1409 to 1417 cm^−1^, showing the formation of an alginate-Ca structure by ionic bonding between carboxyl groups of alginate and Ca^2+^. After the addition of CaCl_2_, the stretching vibration peak of C-O of the alginate that appeared at 1266 cm^-l^ was attributed to the reaction between carboxyl and Ca ion (“C-O-Ca-O-CO-” group structure), which enhanced C-O vibration [5]. These changes indicate that the Ca ion formed an “egg tray” structure with the sodium alginate molecular chain.

The absorption band at 1081 cm^−1^ relating to the C-C and C-O stretching can be also attributed to the presence of cross-linking. The peak of C-C stretching (1030 cm^−1^) shows a higher intensity, suggesting either a stronger O-H binding vibration or a stronger binding of the Ca^2+^ to the guluronic acids from sodium alginate chains. Moreover, the stretching vibration bands observed at approximately 941 cm^−1^, 891 cm^−1^, and 819 cm^−1^ were specifics to the guluronic and mannuronic acids from sodium alginate chains [6].

### 3.3. Effect of Crosslinking Reaction on Microstructure of New Composite Foam Materials with Microscaffolds

**Figure 4:**
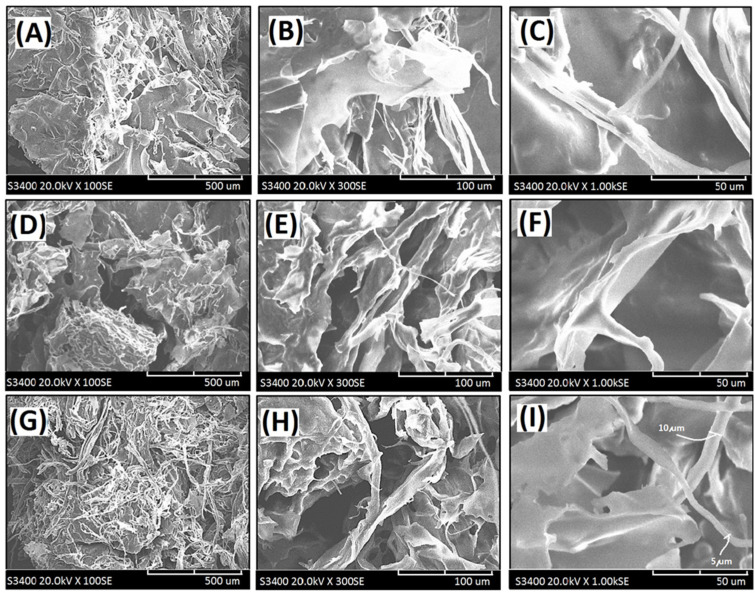
Scanning electron micrographs of the cross-linked composite foam materials (CFM) with decellularized elastic cartilage microscaffolds: (**A**) PCFM1N (100×), (**B**) PCFM1N (300X), (**C**) PCFM1N (1000×), (**D**) PCFM2N (100×), (**E**) PCFM2N (300×), (**F**) PCFM2N (1000×), (**G**) PCFM3N (100×), (**H**) PCFM3N (300×), and (**I**) PCFM3N (1000×).

**Figure 5:**
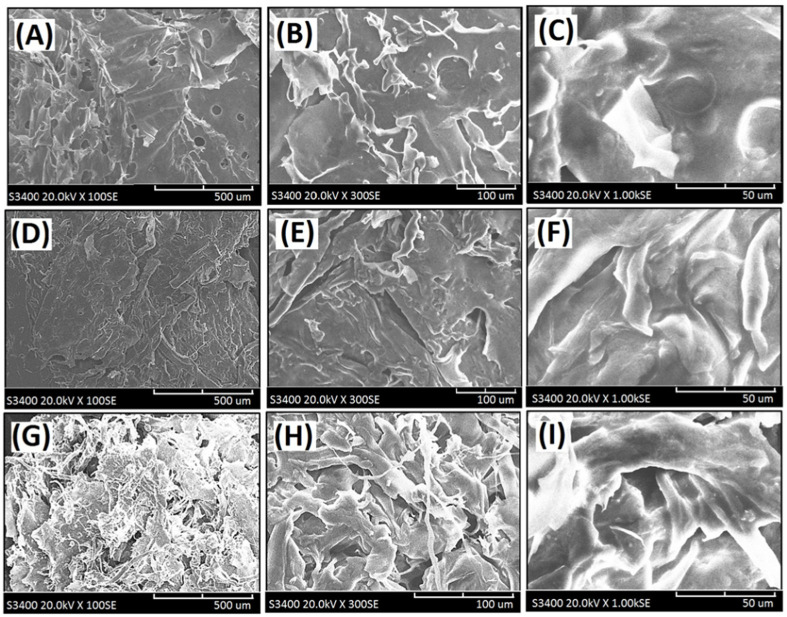
Scanning electron micrographs of the cross-linked composite foam materials with decellularized elastic cartilage microscaffolds: (**A**) PCFM1H (100×), (**B**) PCFM1H (300×), (**C**) PCFM1H (1000×), (**D**) PCFM2H (100×), (**E**) PCFM2H (300×), (**F**) PCFM2H (1000×), (**G**) PCFM3H (100×), (**H**) PCFM3H (300×), and (**I**) PCFM3H (1000×).

The microstructures of the resulting crosslinked composite foam materials with collagen scaffolds were characterized by scanning electron microscope (SEM), as shown in Figure 4 and Figure 5.

**Figure 6:**
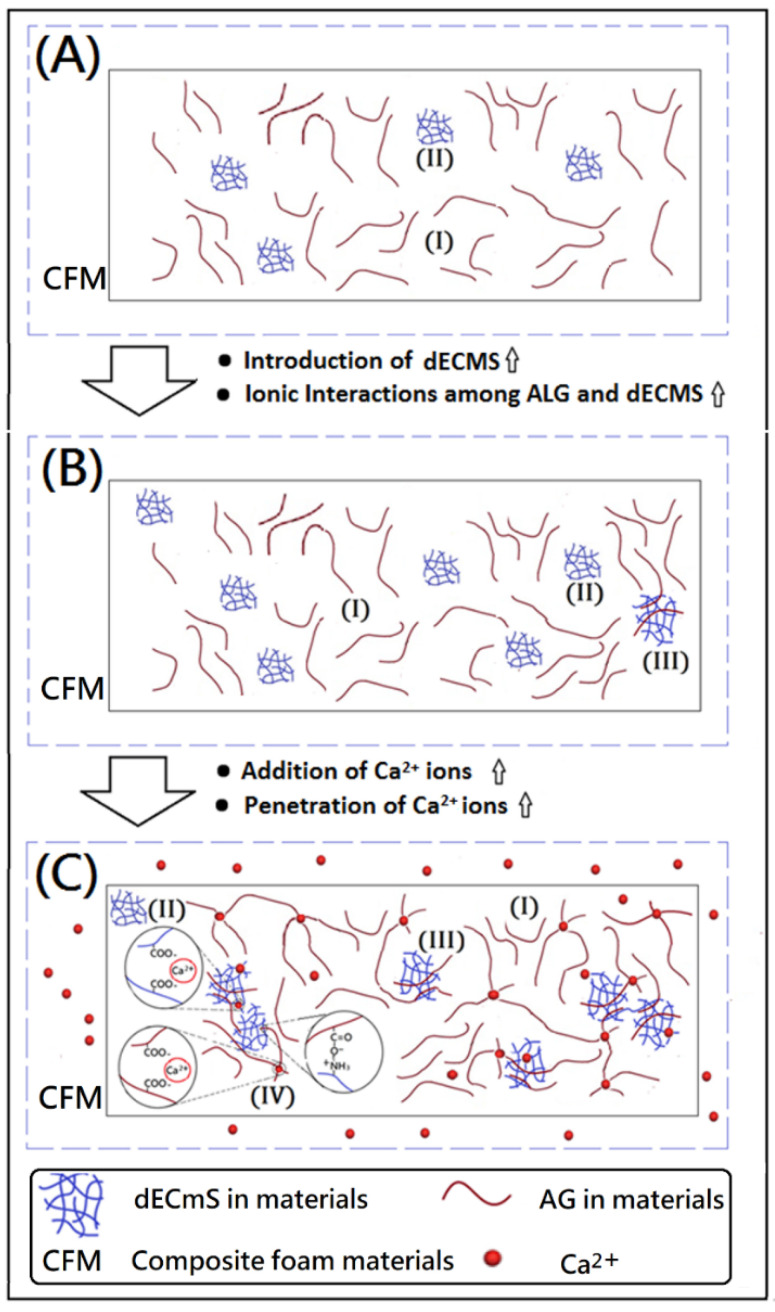
Proposed model of ionic associations among Ca^2+^ ions, AG molecules, and dECmS molecules within designed composite foam materials (CFM): (**A**) new composite foam materials with a small amount of dECmS, (**B**) new composite foam materials with a large amount of dECmS, and (**C**) new cross-linked composite foam materials with a large amount of dECmS.

The loosened scaffold with a thin sheet microstructure from AG with a wide range of 1–2 µm was found in PCFM1N, as shown in Figure 4A–C. Furthermore, scanning electron micrographs of resulting composite foam materials with dECmS scaffold after different introduction ratios are shown in Figure 4D–I. With increasing introduction ratios of dECmS/AG, the remarkable loosened porous microscaffold structures with several fibrous microstructures were observed. Most of the area of the composite foam materials showed a porous microstructure from AG molecules without the addition of CaCl_2_, which showed a sheet boundary in the loosened porous microstructure, such as microstructure (I) and microstructure (II) (Figure 6A,B). The loosened scaffold with a thin sheet microstructure from AG and some fibrous microstructures from dECmS with a wide range of 5–10 µm was found in PCFM3N, as shown in Figure 4G–I. The merged structures that combined the sheet structure of AG molecules with the microstructure of dECmS molecules were observed in PCFM1H with relative high incorporation of AG molecules. Similar behaviors were observed in alginate-based composite scaffold membranes containing decellularized porcine skin [23].

A high amount of dECmS microscaffold enhanced some associations among AG molecules and dECmS molecules. A complicated microstructure (III) formed in the microstructures of the resulting crosslinked composite foam materials, which might further provide a relatively strong structural stability and high thermal stability, as shown in Figure 6B. Additionally, Marangoni Júnior reported that incorporating hydrolyzed collagen into alginate film could increase the maximum degradation rate temperature from 226.51 to 232.89 °C [27]. In this study, the composite foam materials containing dECmS microscaffolds and corresponding cross-linked composite foam materials containing dECmS microscaffolds showed relatively high thermal stabilities (T_dmax_ > 300 °C). However, SEM images showed that the addition of dECmS microscaffold led to a discontinuity in the microstructure of the resulting composite foam materials. The discontinuity in the microstructures was also observed in the alginate-based composite scaffold membrane with high incorporation of decellularized porcine skin [23] and the hydrolyzed collagen/alginate composite matrix with high incorporation of hydrolyzed collagen [27].

Furthermore, crosslinking reactions were carried out because of the addition of CaCl_2_, and the fibrous microstructures merged in the structure of the resulting crosslinked composite foam materials from AG, and a relatively compacted shape was remarkably formed, as shown in Figure 5A–I. It might be due to several associations within the microstructures of composite foam materials containing some fibrous microstructures from dECmS and thin sheet microstructure from AG. With the addition of CaCl_2_, crosslinking reactions provided a relatively complicated microenvironment in the presence of CaCl_2_, which is proposed in the schematic diagrams shown in Figure 6. When Ca^2+^ was introduced and penetrated into the composite foam material in a short period of time, the ionic association between -COO- group of the AG molecule and the -NH^3+^ group of the dECmS molecule and ionic associations among the -COO- group of the AG molecule, Ca^2+^, and the -COO- group of the AG molecule enhanced structural stability. Similarly, Chen et al. reported the cross-linked interpenetrating algainte/gelatin material demonstrated super structural stabilities compared with semi-interpenetrating algainte/gelatin material [25].

Some area of composite foam materials showed smooth morphology in the microstructure, which might have been due to dECmS segments merging into the AG segments and the mixed complex associations among AG segments, dECmS segments, and Ca^2+^ ions. The mixed complex associations among AG segments, dECmS segments, and Ca^2+^ ions introduced complex microstructures, such as microstructure (III) and microstructure (IV), as shown in Figure 6C. When a large amount of dECmS was introduced and Ca^2+^ ions was penetrated into the composite foam material, several kinds of ionic associations such as ionic association between the -COOH group of AG molecule and the -NH_2_ group of the dECmS molecule, ionic associations between the -COOH group of the ALG molecule, Ca^2+^, and the -COOH group of the dECmS molecule, and ionic associations between the -COOH group of the dECmS molecule, Ca^2+^, and the -COOH group of the dECmS molecule easily built up a complicated microstructure. Most areas of composite foam materials showed smooth morphology in the continuous microstructure (Figure 5G–I), which might provide a strong structural stability and a high thermal stability of new crosslinked composite foam materials containing AG segments, dECmS segments, and Ca^2+^ ions. It might be a good potential material as a bioink for medical applications. When a small amount of dECmS was introduced and Ca^2+^ ions were penetrated into the composite foam material, a similar behavior was observed. However, the small amount of dECmS provided a relatively weak association and a relatively smooth morphology was observed (Figure 5A–C).

### 3.4. Effect of Crosslinking Reaction on Thermal Stability of Composite Foam Materials with Microscaffolds

**Figure 7:**
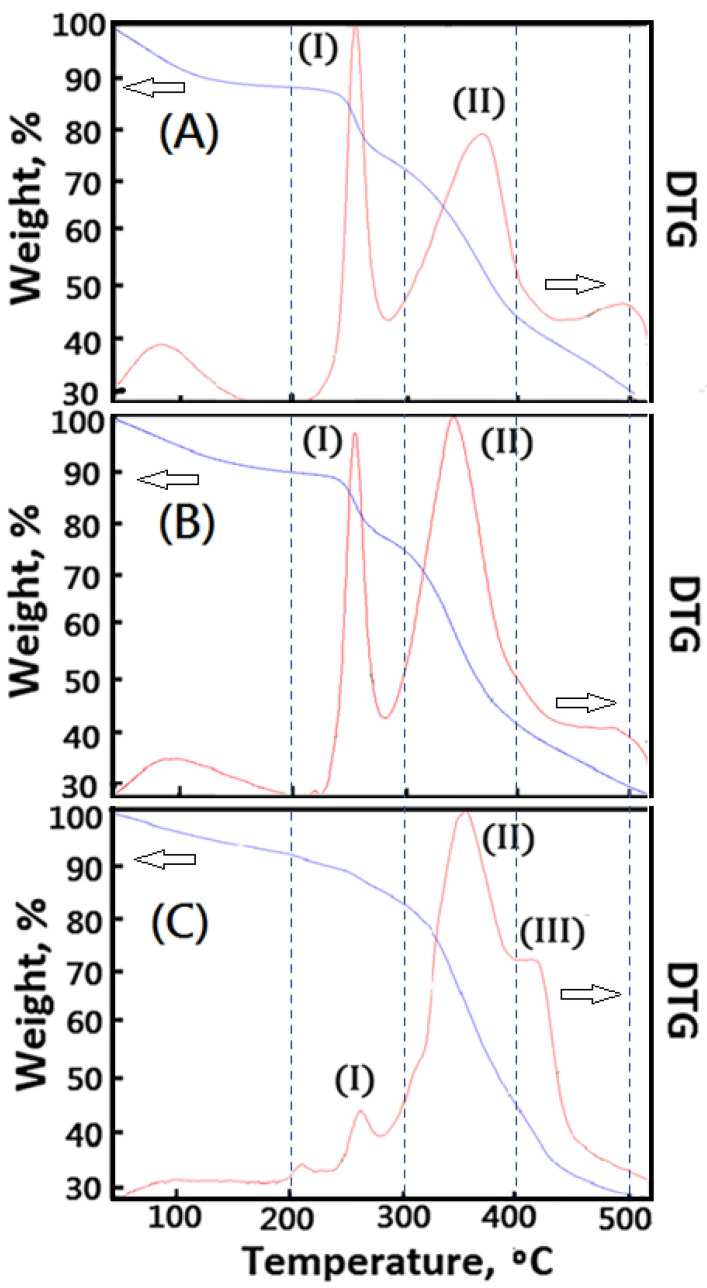
Thermogravimetric analysis of the composite foam materials without CaCl_2(aq)_: (**A**) PCFM1N, (**B**) PCFM2N, and (**C**) PCFM3N.

The thermal stability of 3D bioprinted materials is important. However, AG material shows quite poor thermal stability (Tdmax < 250 °C), which is harmful to being a good carrier for 3D bioprinting medical applications. In order to enhance the thermal stability of the designed polymeric composite foam materials, decellularized elastic cartilage microscaffolds (dECmS) were introduced. The thermal stability of resulting AG-based polymeric composite foam materials containing dECmS was characterized by TGA. In this study, DTG curves were useful for studying the effect of crosslinking functionality on microstructure and thermal properties of new polymeric composite foam materials containing dECmS with different introduction ratios of dECmS/AG. The effect of crosslinking functionality on thermal properties of the new polymeric composite foam materials containing decellularized elastic cartilage microscaffold, dECmS, with various introduction ratios of dECmS/AG was studied. A series of cross-linked polymeric composite foam materials containing dECmS was obtained. A relatively higher maximum pyrolysis temperature (Tdmax) of the resulting composite foam materials containing dECmS than 249 degrees was obtained because of the introduction of dECmS molecules into the resulting AG-based polymeric composite foam materials (Figure 7). The resulting composite foam materials containing dECmS were a good heat-resistant material.

The peaks of DTA curves were considered as some formations of new microstructures, which showed different maximum pyrolysis temperatures in the different stages of temperature, such as the stage of 50–200 °C, the stage of 200–300 °C, the stage of 300–450 °C, and the stage of 450–500 °C. When large amounts of dECmS were introduced into the composite foam material (dECmS/AG = 20/80), a complicated microstructure (III) combining AG molecules with dECmS molecules formed and was observed in the results of DTA analysis. A maximum pyrolysis temperature of 420 °C was observed, as shown in Figure 7C. The DTA peak of microstructure (I) of AG molecules shifted to a higher Tdmax value than 260 °C, which contributed to the interaction of AG molecules and dECmS molecules. Similarly, the DTA peak of microstructure (II) shifted to a higher Tdmax value than 360 °C, which contributed to the interaction of dECmS molecules and AG molecules. When slight amounts of dECmS were introduced into the polymeric composite foam materials (dECmS/AG = 5/95), a complicated microstructure (III) combining AG molecules with dECmS molecules was not formed in the results of DTA analysis, as shown in Figure 6A. The DTA peaks of AG molecules’ microstructure (I) and dECmS molecules’ microstructure (II) was observed at 250 °C and 370 °C, respectively. The DTA peak of microstructure (II) shifted to a higher Tdmax value than 370 °C which contributed to the strong interaction of dECmS molecules and AG molecules of the AG-rich composite material. Similarly, when small amounts of dECmS were introduced into the composite material (dECmS/AG = 15/85), the complicated microstructure (III) and the shifted Tdmax value was not observed in the results of DTA analysis, as shown in Figure 7B.

**Figure 8:**
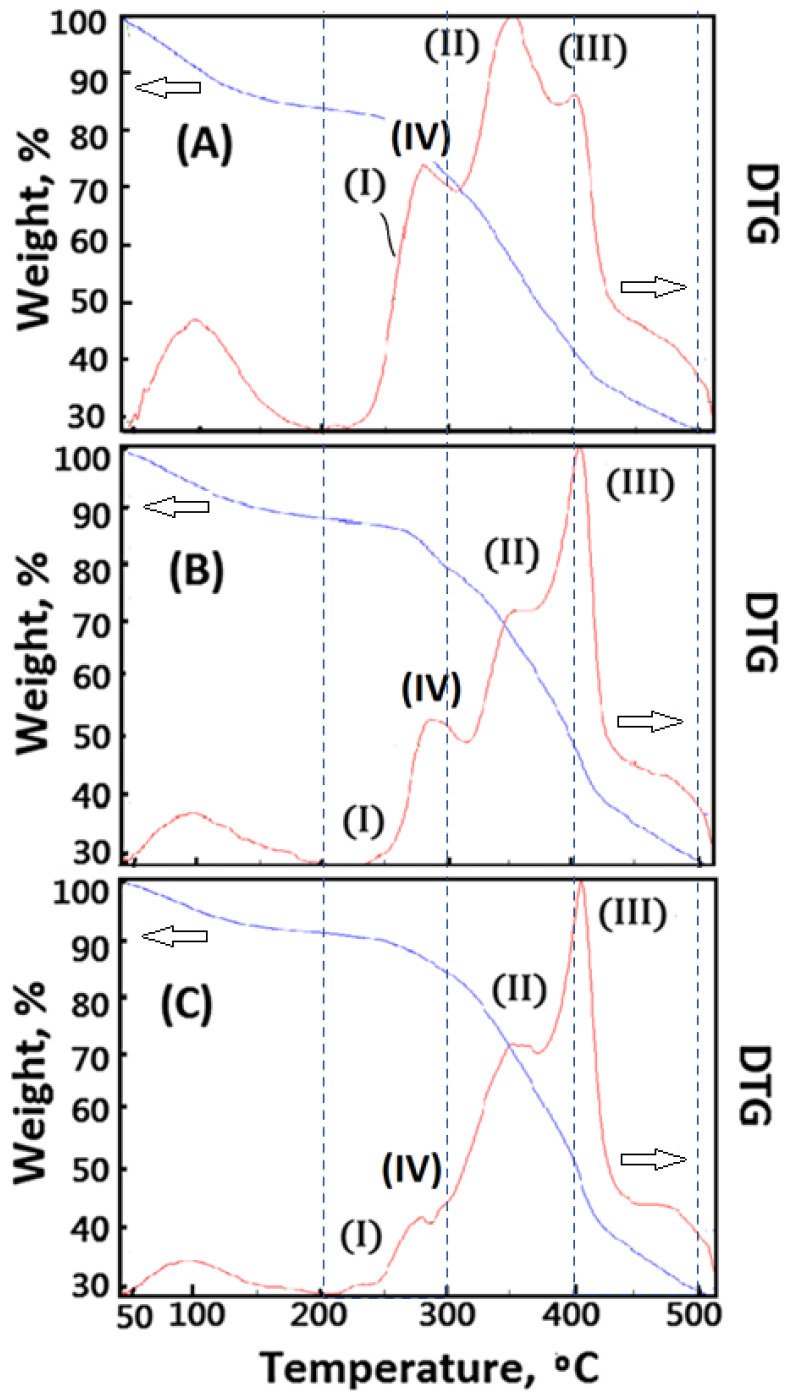
Thermogravimetric analysis of the composite foam materials with CaCl_2(aq)_: (**A**) PCFM1H, (**B**) PCFM2H, and (**C**) PCFM3H.

The composite material was prepared by the lyophilized technology. The resulting composite material was further crosslinked with CaCl_2(aq)_ to obtain cross-linked polymeric composite foam materials. However, the crosslinking functionality affected the microstructures of cross-linked composite foam materials containing dECmS molecules. The Ca^2+^ ions penetrated into the porous microstructures to build up the complicated microstructure (III) and a new ionic crosslinked microstructure (IV) of AG molecules, as shown in Figure 8A–C. When slight amounts of dECmS were introduced into the composite material (dECmS/AG = 5/95), a complicated microstructure (III) combining AG molecules with dECmS molecules was not formed in the results of DTA analysis, as shown in Figure 7A. The DTA peaks of AG molecules’ microstructure (I) and dECmS molecules’ microstructure (II) was observed at 250 °C and 370 °C, respectively. The DTA peak of microstructure (II) shifted to a higher Tdmax value than 370 °C which might have contributed to the strong interaction of dECmS molecules and AG molecules of the AG-rich composite material. When Ca^2+^ ions were introduced into the AG-rich composite material (dECmS/AG = 5/95), the complicated microstructure (III) and the ionic cross-linked microstructure (IV) was enhanced, as shown in Figure 8A. Good thermal stability of PCFM1H was observed. The designed cross-linked AG-based composite foam materials containing dECmS and Ca^2+^ ions with good thermal stabilities was considered to be a good heat-resistant composite material for medical and bioprinting applications.

## 4. Conclusions

In this study, a series of new polymeric composite foam materials containing decellularized elastic cartilage microscaffolds, dECmS, were successfully obtained from alginate and porcine elastic cartilage by using supercritical carbon dioxide fluid technology and papain treatments. The retained extra-cellular matrix and integrity of the scaffold-structure were observed in the resulting polymeric composite foam materials. This work provides a simple and time-saving procedure for preparing composite foam materials with decellularized elastic cartilage microscaffolds, which show a microstructure with enhanced thermal stability. The effect of cross-linking functionality on microstructures and thermal properties of new polymeric composite foam materials containing dECmS with various dECmS/AG ratios was studied. Furthermore, the microstructures of the cross-linked composite foam materials containing dECmS were observed, defined, and studied, depending on their specific associations and DTG results. The Tdmax of the resulting polymeric composite foam materials containing dECmS molecules and Ca^2+^ ions was increased. Polymeric composite foam materials with good thermal stability were successfully obtained. The resulting polymeric composite foam materials with fibrous microscaffolds could be considered as a 3D bioprinting material or a bioink for medical applications. The properties, depending on compositions of dECmS and AG, could provide a good parameter for bioinks and 3D bioprinting applications.

## Figures and Tables

**Table 1:** Preparation of new composite foam materials with decellularized elastic cartilage microscaffolds.

Sample No.	dECmS/AG ^(a)^ (*w/w*)	[CaCl_2_]
PCFM0N	100/0	-
PCFM1N	5/95	-
PCFM2N	15/85	-
PCFM3N	20/80	-
PCFM1H	5/95	5%
PCFM2H	15/85	5%
PCFM3H	20/80	5%

^(a)^ AG, alginate, and dECmS, decellularized elastic cartilage microscaffold, via ScCO_2_ treatments.

## Data Availability

Not applicable.

## References

[1] Liaw D.J., Huang C.C., Lee W.F., Borbély J., Kang E.T. (1997). Synthesis and Characteristics of the Poly(carboxybetaine)s and the Corresponding Cationic Polymers. J. Polym. Sci. Part A Polym. Chem..

[2] Liu Y.W., Huang C.C., Wang Y.Y., Xu J., Wang G.D., Bai X.P. (2021). Biological Evaluations of Decellularized Extracellular Matrix Collagen Microparticles Prepared Based on Plant Enzymes and Aqueous Two-phase Method. Regen. Biomater..

[3] Vidal B.C., Mello M.L.S. (2016). FT-IR Microspectroscopy of Rat Ear Cartilage. PLoS ONE.

[4] Mata-Miranda M.M., Martinez-Cuazitl A., Guerrero-Robles C.I., Noriega-Gonzalez J.E., Garcia-Hernandez J.S., Vazquez-Zapien G.J. (2019). Biochemical similarity between cultured chondrocytes and in situ chondrocytes by chemometric analysis from FTIR microspectroscopy. Biotechnol. Rep..

[5] Ribeiro C.C., Barrias C.C., Barbosa M.A. (2004). Calcium phosphate-alginate microspheres as enzyme delivery matrices. Biomaterials.

[6] Fernandes R.S., Moura M.R., Glenn G.M., Aouada F.A. (2018). Thermal, microstructural, and spectroscopic analysis of Ca2+ alginate/clay nanocomposite hydrogel beads. J. Mol. Liq..

[7] Liaw D.J., Chen T.P., Huang C.C. (2005). Self-Assembly Aggregation of Highly Stable Copolynorbornenes with Amphiphilic Architecture via Ring-Opening Metathesis Polymerization. Macromolecules.

[8] Liaw D.J., Huang C.C. (1997). Dilute Solution Properties of Poly(3-dimethyl acryloyloxyethyl ammonium propiolactone). Polymer.

[9] Liaw D.J., Huang C.C., Sang H.C., Kang E.T. (1999). Intramolecular Hydrophobic Aggregation of Amphiphilic Polysulfobetaine with Various Hydrophobic Groups in Aqueous Solution. Langmuir.

[10] Li Z.F., Kang E.T., Neoh K.G., Tan K.L., Huang C.C., Liaw D.J. (1997). Surface Structures and Adhesive-Free Adhesion Characteristics of Polyaniline Films after Modification by Graft Copolymerization. Macromolecules.

[11] Zhai G., Toh S.C., Tan W.L., Kang E.T., Neoh K.G., Huang C.C., Liaw D.J. (2003). Poly(vinylidene fluoride) with Grafted Zwitterionic Polymer Side Chains for Electrolyte-Responsive Microfiltration Membranes. Langmuir.

[12] Racovita S., Trofin M.-A., Loghin D.F., Zaharia M.-M., Bucatariu F., Mihai M., Vasiliu S. (2021). Polybetaines in Biomedical Applications. Int. J. Mol. Sci..

[13] Smidsrød O., Skjak-Braek G. (1990). Alginate as Immobilization Matrix for Cells. Trends Biotechnol..

[14] Pagano S., Lombardo G., Costanzi E., Balloni S., Bruscoli S., Flamini S., Coniglio M., Valenti C., Cianetti S., Marinucci L. (2021). Morpho-functional effects of different universal dental adhesives on human gingival fibroblasts: An in vitro study. Odontology.

[15] Lee K.Y., Mooney D.J. (2012). Alginate: Properties and biomedical applications. Prog. Polym. Sci..

[16] Anal A.K., Stevens W.F. (2005). Chitosan-alginate multilayer beads for controlled release of ampicillin. Int. J. Pharm..

[17] Liu D., Zhao K., Qi M., Li S., Xu G., Wei J., He X. (2018). Preparation of Protein Molecular-Imprinted Polysiloxane Membrane Using Calcium Alginate Film as Matrix and Its Application for Cell Culture. Polymers.

[18] Huang C.C., Liu C.Y., Huang C.Y., Liu H.W. (2014). Carbodiimide Crosslinked and Biodegradation-controllable Small Intestinal Submucosa Sheets. J. Bio-Med. Mater. Eng..

[19] Camacho N.P., West P., Torzilli P.A., Mendelsohn R. (2001). FTIR microscopic imaging of collagen and proteoglycan in bovine cartilage. Biopolymers.

[20] Vogel K.G., Trotter J.A. (1987). The Effect of Proteoglycans on the Morphology of Collagen Fibrils Formed In Vitro. Collagen Relat. Res..

[21] Theocharis A.D., Skandalis S.S., Gialeli C., Karamanos N.K. (2016). Extracellular matrix structure. Adv. Drug Deliv. Rev..

[22] Lee J., Hong J., Kim W., Kim G.H. (2020). Bone-derived dECM/alginate bioink for fabricating a 3D cell-laden mesh structure for bone tissue engineering. Carbohydr. Polym..

[23] Huang C.C. (2021). Characteristics and Preparation of Designed Alginate-Based Composite Scaffold Membranes with Decellularized Fibrous Micro-Scaffold Structures from Porcine Skin. Polymers.

[24] Huang C.-C., Chen Y.-J., Liu H.-W. (2021). Characterization of Composite Nano-Bioscaffolds Based on Collagen and Supercritical Fluids-Assisted Decellularized Fibrous Extracellular Matrix. Polymers.

[25] Marangoni Júnior L., Rodrigues P.R., da Silva R.G. (2021). Sustainable Packaging Films Composed of Sodium Alginate and Hydrolyzed Collagen: Preparation and Characterization. Food Bioprocess Technol..

[26] Chen Q., Tian X., Fan J., Tong H., Ao Q., Wang X. (2020). An Interpenetrating Alginate/Gelatin Network for Three-Dimensional (3D) Cell Cultures and Organ Bioprinting. Molecules.

[27] Choi Y.C., Choi J.S., Kim B.S., Kim J.D., Yoon H.I., Cho Y.W. (2012). Decellularized extracellular matrix derived from porcine adipose tissue as a xenogeneic biomaterial for tissue engineering. Tissue Eng. Part C Methods.

